# Three-Dimensional Models of Soil-Transmitted Helminth Eggs from Light Microscopy Images

**DOI:** 10.3390/tropicalmed7090216

**Published:** 2022-08-30

**Authors:** Yan Emygdio Dias, Elisângela Oliveira de Freitas, Dayane Alvarinho de Oliveira, Wendell Girard-Dias, Lúcio Paulo do Amaral Crivano Machado, Eduardo José Lopes-Torres

**Affiliations:** 1 Laboratório de Helmintologia Romero Lascasas Porto, Departamento de Microbiologia, Imunologia e Parasitologia, Faculdade de Ciências Médicas—Universidade do Estado do Rio de Janeiro, Rio de Janeiro 20550-170, Brazil; 2 Instituto Oswaldo Cruz, Fundação Oswaldo Cruz, Rio de Janeiro 21040-900, Brazil; 3 Centro Universitário Universus Veritas, Rio de Janeiro 22230-060, Brazil; 4 Departamento de Ensino de Ciências e Biologia, Instituto de Biologia Roberto Alcantara Gomes-UERJ, Rio de Janeiro 20511-010, Brazil

**Keywords:** helminth, 3D printing, health education, *Trichuris muris*, *Ascaris lumbricoides*, science teaching

## Abstract

The World Health Organization indicates that more than 1.5 billion people are infected with geohelminths. Soil-transmitted helminths prevail mostly in tropical and subtropical regions, in areas with inadequate hygiene and sanitation conditions, and basic health education problems. Nematode eggs are structures of resistance and infection by fecal–oral transmission. When STH eggs are ingested, they can infect the potential host, causing abdominal pain, diarrhea, anemia, malnutrition, and physical-cognitive impacts in children. Taking advantage of the increasing employment of three-dimensional models of these structured based on light microscopy images to improve the research area and education could be an alternative to improve health education and spread scientific information on transmission and prevention. The objective of this work was to produce 3D printed models from bi-dimensional images of eggs based on their real morphological and morphometric characteristics. The virtual models were reconstructed from the acquisition and selection of images obtained using light microscopy. After selecting referential images, we constructed the models based on the vectorization of the egg structures. After vectorization, 3D modeling was performed and printed in PLA. 3D models have a high potential to contribute to the advanced morphological studies and teaching of parasitological sciences, enriching the teaching-learning process applicable in presential or remote teaching of basic education, undergraduate, and post-graduation classes.

## 1. Introduction

Intestinal parasite infections are serious public health problems, especially in developing tropical nations due to the precarious conditions of basic sanitation. They are among the pathogens that most cause morbidity and mortality in immunocompromised people and children [1]. Worms worldwide infect more than 1.5 billion people (24% of the population). These are neglected diseases, with prevalence related to socio-economic aspects, impacting poor people, mainly children, and aggravating social inequality [2].

Soil-transmitted helminths (STH) infections are caused by nematodes that develop in the soil (eggs or larvae). The most common symptoms of STH infection in school-age children are abdominal pain, diarrhea, and, in severe cases, anemia and malnutrition, impacting their general development at a critical age. In tropical and subtropical areas, climate and sanitary conditions enable the transmission of STH [3], and according to WHO data, a large part of the world population, mainly school-age children, is infected with a broad spectrum of parasitic protozoa and helminths [4]. School-age children are more susceptible to parasitic diseases because of the maturation of the immune system and precarious hygiene habits, becoming more susceptible to infections and reinfections [4,5]. A single administration dose of mebendazole is the treatment in Brazil. However, STH presents a high research prevalence, mainly in ascariasis and trichuriasis, and drug resistance for *Trichuris trichiura* has been reported [6,7]. The constant intestinal parasitism in children, including infections during pregnancy, affects the physical, psychosomatic, and social development of school-age children [8,9]. 

The resistance of the egg structure contributes to soil dispersion, amplifying the infection impact and identification in human coprolites [10,11]. The nematode eggshell is a complex structure with characteristics that promote impermeability, protecting the larvae parasite from osmotic stress and providing stable physiological conditions [12]. *Trichuris muris* is a powerful model for *T*. *trichiura*, as it is a natural parasite of wild mice. The eggs, larvae, and adult worms demonstrate similar morphology to *T*. *trichiura*. Human whipworm eggs are ellipsoidal and have a characteristic barrel-shape that measures 57–78 µm in length and 26–30 µm in width [13] and two polar plugs, enabling the L1 larvae to hatch. Roundworm eggs are oval to round and have a characteristic outer mamillated layer, measuring 45–75 μm in length and 35–50 μm in width [14].

Biological information on parasites and their structures is important to improve knowledge about transmission and control, working as a complementary strategy to prevent infections. The study of helminths and the description of their structures, including eggs, are based on two-dimensional images. This information describes diagnostic characteristics such as the topographic interpretation of the outer layer of *Ascaris lumbricoides* eggs as mamillated. Three-dimensional models can promote the tactile experience in learning parasitology, improving new methodologies in health education projects and innovative tools to assist the orientation in hygiene behavior in the face of these diseases [15]. From this perspective, these models can facilitate the teaching-learning process, favoring a more dynamic environment with more active and collaborative student participation from elementary school to the higher education of health professionals [16,17], including the education of the visually impaired. 

The use of new technologies in medical education was spread via remote systems during the COVID-19 pandemic, and was made possible by tablets, cell phones, or computers, improving access to health information for poor communities. However, even before the pandemic, the use of digital technologies in health and science education, including the use of three-dimensional modeling, 3D printers [18,19], and 3D microscopy, was a clear trend with challenges, especially for tropical developing countries. 

In the present work, we build 3D-printed models of soil-transmitted helminth eggs (*Trichuris muris* and *Ascaris lumbricoides*) based on 2D light microscopy images, suggesting these models to improve parasitology teaching in health education projects and health professional formation. To enable the use of this technology by developing countries, we performed the virtual and printed model processes using free and open-source software.

## 2. Materials and Methods

### 2.1. Light Microscopy of the Nematode Eggs

The nematode eggs were recovered from parasite experimental life cycles maintained in Swiss mice at the Laboratório de Helmintologia Romero Lascasas Porto—State University of Rio de Janeiro and the Laboratório de Imunologia e Genômica de Parasitos—Federal University of Minas Gerais, and all the animal protocols were approved by the Ethics Committee for Animal Experimentation under protocol numbers UERJ 020/2018 and UFMG 54/2012, according to Brazilian federal law (Law 11.794/2008, regulated by Decree 6.899/2009).

The eggs were chemically fixed by immersion in 4% paraformaldehyde (EMS^®^) in 0.1M cacodylate buffer overnight, washed in buffered saline (PBS) three times for 15 min, and mounted in temporary slides with PBS. Twenty eggs (each sample: *Trichuris muris* and *Ascaris lumbricoides*) were analyzed using a Nikon Eclipse 80i microscope with a differential interference contrast (DIC) system, and the images were obtained using the digital camera Nikon DS-Ri1.

### 2.2. Three-Dimensional Virtual Modeling

Based on 2D light microscopy images, we identified and selected the egg showing the structures of interest with highest contrast (eggshell and larvae details) and started the vectorization using the Inkscape software, which consists in manually selecting the area or structure of interest by freehand tracing and converting it to a vector representation of the image. Details of the *Trichuris muris* and *Ascaris lumbricoides* eggs were separately vectored to be exported to the free Tinkercad^®^ website. The vectorized two-dimensional segments were converted into three-dimensional parts associated with the egg structures and identified with different colors. To improve details of the model based on the egg structures observed by microscopy, we used Inkscape (The Inkscape team), 3D Builder (Microsoft^®^), applied to correct small errors, and Sculptris used to enable the finalization of the model with the creation of an external eggshell structure. We used one image of the egg, carefully selected for each species, for the final virtual model. 

### 2.3. Three-Dimensional Printing

Virtual models were exported in STL and converted (slicing) for printing extension through the Autodesk Cura program. Models were printed using the Creality Ender 3 and Tevo Tarantula Pro machines with the following parameters: 0.2 mm layer thickness, 1.2 mm wall thickness, and 10% fill. The nozzle temperature was kept at 200 °C, the print bed temperature at 60 °C, and the print speed was 50 mm/s. The printing was performed using the Fused Filament Fabrication (FFF) technique. The Polylactic Acid filament (PLA) is heated through an extrusion mechanism, consisting of a heat sink, a resistor, a block heating, and a printing nozzle. The molten plastic is deposited on a flat surface of the printer, layer after layer, to create the three-dimensional model.

## 3. Results

**Figure 1:**
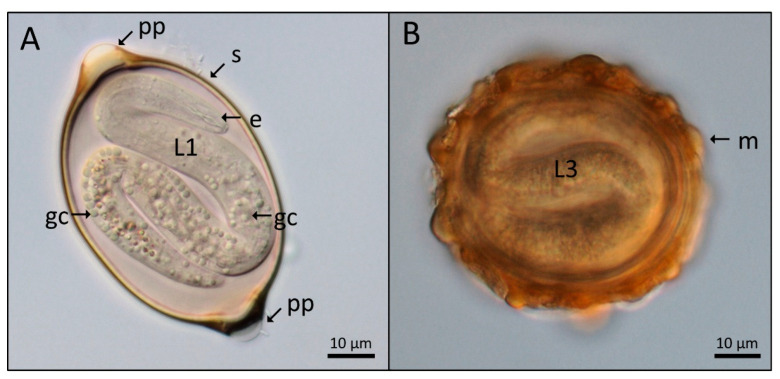
Light microscopy (DIC) of nematode eggs. (**A**): *Trichuris muris* egg showing in the eggshell (s) extremities two polar plugs (pp). Inside the egg are the first-stage larvae (L1), with the esophagus (e) and germinative cells (gc). (**B**): *Ascaris lumbricoides* egg showing the external mamillated layer (m) and the third-stage larvae (L3).

Imagens obtained by light microscopy of the embryonated eggs of *Trichuris muris* and *Ascaris lumbricoides* were selected based on details of the eggshell structure and larvae morphology. Embryonated *T. muris* egg image showed the general morphology of trichurids, highlighting eggshell and L1 larvae structures, such as polar plugs (operculum) and three major eggshell layers (yolk, chitin, and lipid layers). In the larvae, it is possible to identify the outline of the whole body, esophagus, and germ cells (Figure 1A). The same criteria were used to select the fertile *A. lumbricoides* egg, showing the characteristic oval to round shape (almost spherical), outer mamillated layer eggshell, and the outline of the L3 larvae body (Figure 1B).

**Figure 2:**
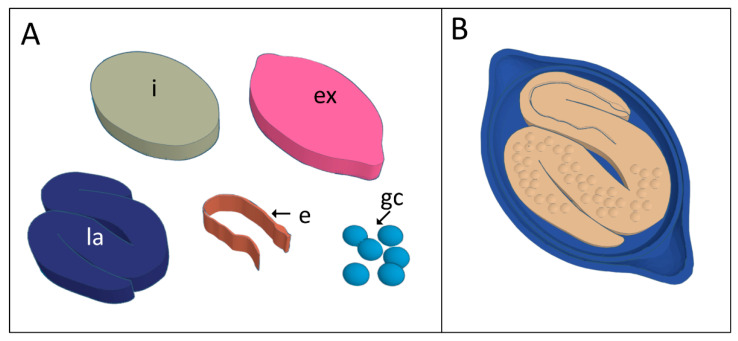
Three-dimensional virtual models of the different parts of the *Trichuris muris* egg obtained using the Tinkercad website. (**A**): Internal area of the egg (i), external layer (ex), larvae (la), esophagus (e), and germinative cells (gc). (**B**): Virtual model of a complete *T. muris* egg integrating all the different parts shown in Figure 2A.

Selected images were exported in .tiff to Inkscape program, in which they went through an automatic vectorization process through the Trace Bitmap tool, which enabled a pre-vectorization. Segments selected of interest structures *T. muris* egg: three eggshell layers, polar plugs, and anterior (esophageal tube) and posterior regions (germ cells) of the larvae; and in *A. lumbricoides* egg: mamillated layer, and the L3 larvae body. The images Scalable Vector Graphics (.svg) extension were exported from Inkscape to Tinkercad^®^ website for conversion to three-dimensional volumes. All structures were converted directly by the software using the vectors obtained from the microscopy images; only for the germ cells that we used a spherical shape tool built based on the size of the cells observed on the *T. muris* larvae (Figure 2A).

Final models were built in Tinkercad^®^ using the addition and exclusion tools. *Trichuris muris* egg model construction was performed with the distribution of germ cells in the posterior region of the larvae, reproducing the microscopy image. The esophageal tube was inserted in the larvae using the structure’s contour to exclude a part of the larvae volume, creating a groove in bas-relief representing this primordial digestive structure in the anterior region. For the three eggshell layers formation, we duplicate the image of the external layer to have a background and a continuous layer including the polar parts, forming the two polar plugs (Figure 2B). For *A. lumbricoides* modeling, we excluded the inner part of the egg, using the limit of the inner layer of the eggshell, generating a depth to fit L3 larvae. 

**Figure 3:**
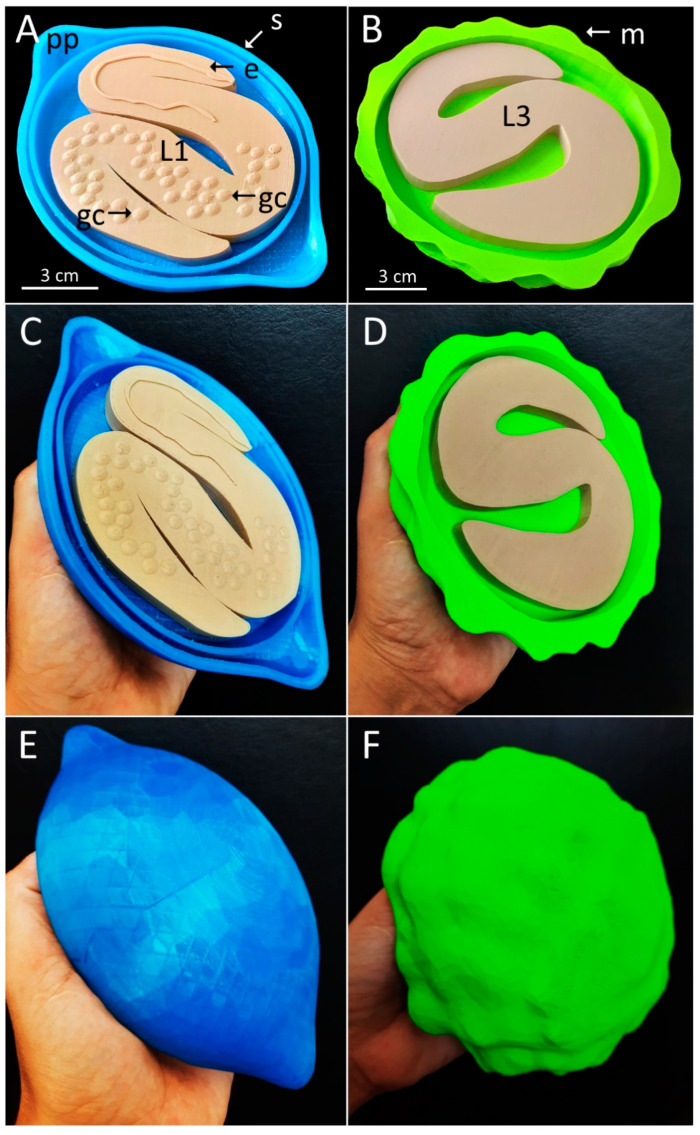
Photographs showing different sides of the printed egg models. (**A**,**C**,**E**): *Trichuris muris* egg (blue) measuring 20 cm × 12 cm, showing the polar plugs (pp), smooth eggshell (s), first-stage larvae (L1), esophagus (e), and germ cells (gc). (**B**,**D**,**F**): *Ascaris lumbricoides* egg (green) measuring 17 cm in diameter, showing third-stage larvae (L3) and the texture of the mamillated layer (m) on the outer surface of the egg model.

External eggshell shape and texture is an important characteristic to differentiate the STH eggs. Based on the model size and morphology constructed grounded on microscopy images, we used a manual modeling tool, Sculptris^®^, to create a three-dimensional eggshell in freely sculpting. Using the digital sculpture, we create a smooth texture with polar plugs contours for *T. muris* egg and the mamillated external layer for *A. lumbricoides* (Figure 3A,B). These models were exported in the .obj format to the Tinkercad^®^ platform, and were resized, grouped, and adjusted to the corresponding proportional shape of each egg. Before printing, size proportionality was calculated using the real measures of the two eggs. These models were exported in .stl (Standard Triangle Language) format to Ultimaker Cura software, processed by “slicing,” divided into layers, and finally exported into an instruction file for printing. The parts of the models were printed using different colors; the pieces (larvae and eggshell) were mounted after being printed separately. 

The eggs were printed in models magnified 2850 times the original egg size, resulting in the *T. muris* model with 20 cm (158g) and the model of *A. lumbricoides* with 17 cm (175 g). The cost reference was 20 USD per kilogram of Polylactic Acid (PLA), approximately 3 USD for the *T. muris* egg, and 3.35 USD for the *A. lumbricoides* egg model. The eggshell’s printing time was approximately 12 h and the larvae 6 h for each model (Figure 3C–F).

## 4. Discussion

The use of three-dimensional models, educational games and other tools for pedagogical practices can develop significant learning, playfully build important knowledge to strengthen responsibilities in relation to individual and collective health, and enable the development of scientific knowledge [20]. The use of 3D models is an effective method to support learning [21] and we need to expand the use of pathogen models in medical education. These models can increase the accessibility of biomedical themes promoting active learning and inclusive teaching [19], breaking barriers found in the teaching of microscopic structures and some limits for blind students. 

Part of the learning difficulty of students’ and interest about the scientific subjects can be explained due to how teaching is transmitted and not constructed, especially when the used concept is disconnected from the students’ reality, working exclusively theoretically, valuing only memorization [22]. When we work directly in communities, with health education projects, abstract concepts and the difficulty of scaling microscopic structures are further barriers for people in learning and incorporating new knowledge. Three-dimensional models can be complex printed objects based on simple biological images obtained by 2D images or 3D microscopy, including light and electron [23] microscopy. Virtual models can be easily diffused to different schools, universities or teaching hospitals for printing in another location, or used directly in their virtual 3D format.

Microorganisms, microscopic structures of pathogens, and ultrastructural organization in cell biology require an important degree of abstraction. The difficulty of constructing these contents using virtual or printed models can be minimized, improving students’ interest and connection with the content. Previous work has shown that the printing of 3D models has helped in the teaching of human anatomy in medical education [24,25]. Another work focused on teaching blood cells, using 2D transmission electron microscopy images, modeling, and three-dimensional printing of neutrophils to improve cell biology teaching [23]. These works show 3D models’ contribution as new tools to improve teaching in different areas of biological and biomedical sciences. Other works have shown that 3D printed map models and 3D miniatures with action simulations using finger gestures help in the learning process of students with visual impairments [26,27].

The use of models presented here in higher education can improve learning from different perspectives, mainly when the high complexity of helminth eggshell ultrastructure is used in taxonomic classification [28] or when egg morphology is altered after anthelmintic treatments [29]. These details can be better understood using 3D models. In addition, these models can help the training of professionals engaged in identifying parasites, allowing the association of the light microscopy observation with the tactile experience during the formation of these microscopists.

## 5. Conclusions

Three-dimensional models made with PLA are easy to print and to clean, present low-cost, are non-toxic, and have high mechanical resistance. We use Polylactic Acid filament, which is a biodegradable thermoplastic, derived from corn starch or sugar cane, which does not shrink in the cooling process, becoming rigid and insoluble in water. Lactide released into the atmosphere during its production is a non-toxic chemical, being a low emitter of Volatile Organic Compounds (VOCs) and Ultrafine Particles (UFPs) compared to other 3D printing filaments [30,31,32,33,34]. While PLA is not recommended for applications that involve high temperatures, such as autoclaving [35], it was shown to be a strong material, with mechanical strength greater than more proven polymers, such as the non-biodegradable Acrylonitrile Butadiene Styrene (ABS) and Polycarbonate (PC) [35]. The durability of the printed models should not be a concern when carried out in good storage condition, with natural protection against high heat and the weather. The handling for creating the virtual models was through the access to free and open access software, enabling more easily modeling and printing anywhere. Biological 3D-printed models can be used as a basis for mass production of identical copies using injection molding, facilitating large-scale distribution and use in different educational systems. The education environment is favorable for building preventive behaviors against intestinal parasites and other transmissible diseases. Students and trained people are disseminators of correct scientific information, creating an important resistance to negation.
